# Chicken Farming in Grassland Increases Environmental Sustainability and Economic Efficiency

**DOI:** 10.1371/journal.pone.0053977

**Published:** 2013-01-23

**Authors:** Meizhen Liu, Bingxue Wang, Colin P. Osborne, Gaoming Jiang

**Affiliations:** 1 The State Key Laboratory of Vegetation and Environment Change, Institute of Botany, The Chinese Academy of Sciences, Beijing, China; 2 Department of Animal and Plant Sciences, University of Sheffield, Sheffield, United Kingdom; Helmholtz Centre for Environmental Research – UFZ, Germany

## Abstract

**Background:**

Grassland degradation caused by overgrazing poses a threat to both animal husbandry and environmental sustainability in most semi-arid areas especially north China. Although the Chinese Government has made huge efforts to restore degraded grasslands, a considerable attempt has unfortunately failed due to an inadequate consideration of economic benefits to local communities.

**Methodology/Principal Findings:**

A controlled field experiment was conducted to test our hypothesis that utilizing natural grasslands as both habitat and feed resources for chickens and replacing the traditional husbandry system with chicken farming would increase environmental sustainability and raise income. Aboveground plant biomass elevated from 25 g m^−2^ for grazing sheep to 84 g m^−2^ for chicken farming. In contrast to the fenced (unstocked) grassland, chicken farming did not significantly decrease aboveground plant biomass, but did increase the root biomass by 60% (p<0.01). Compared with traditional sheep grazing, chicken farming significantly improved soil surface water content (0–10 cm), from 5% to 15%. Chicken farming did not affect the soil bulk density, while the traditional sheep grazing increased the soil bulk density in the 0–10 cm soil layer by 35% of the control (p<0.05). Most importantly, the economic income of local herdsmen has been raised about six times compared with the traditional practice of raising sheep. Ecologically, such an innovative solution allowed large degraded grasslands to naturally regenerate. Grasslands also provided a high quality organic poultry product which could be marketed in big cities.

**Conclusion/Significance:**

Chicken farming is an innovative alternative strategy for increasing environmental sustainability and economic income, rather than a challenge to the traditional nomadic pastoral system. Our approach might be technically applicable to other large degraded grasslands of the world, especially in China.

## Introduction

The implementation of ‘Reform and Opening up’ in 1978 achieved exceptional economic growth in China. However, it simultaneously caused tremendous environmental problems. For instance, assessed by Environmental Performance Index (EPI), China ranked 121^st^ among 163 countries in 2010 [1], despite the fact that a huge government effort has been attempting environmental protection. A number of factors, ecological, socio-economical, demographic and technological, may have influenced environmental performance of the country which has the largest population in the world. Therefore, integrative approaches are urgently required to enable environmental and ecological restoration in China.

In the semi-arid areas of north China, sandstorms rank among the most serious environmental problems, posing threats to both animal husbandry and social sustainability [2]. It is reported that serious sandstorms hitting the capital city Beijing and nearby regions each year originate from three main sources: degraded grasslands, croplands in the steppe region and dried-up lakes in the arid and semi-arid regions [3], [4], [5]. Years of overgrazing have led to remarkable grassland degradation in north China, causing further ecological disasters such as the blooming of insect pests, appearance of sandstorms or light wind-borne dust clouds in China and neighboring countries such as Korea and Japan [6], [7], [8], [9].

Grasslands account for 41% of the total area of China, 3.3 times the size of its croplands [10]. Yet, because of serious land degradation, these vast grasslands cannot presently sustain the number of animals required to support the livelihoods of local families. Land degradation has both inhibited the ecological functioning of grasslands and negatively affected local economic and social development [11], [12]. Currently, the primary productivity of degraded grassland is only 50% that of a healthy grassland ecosystem [13], and these natural grasslands provid merely 20% of the meat for China [11]. The economic return of raising livestock in seriously degraded areas is even negative in some regions of grassland, especially Inner Mongolia [14]. It has been reported that overgrazing has more influence on the plant communities than climate change in these regions [15].

It is imperative to explore an alternative approach to more sustainably utilize grassland resources without causing further land degradation. According to our past 10 years' practice in ecological restoration in the Hunshandake Sandland of Inner Mongolia, we need first to reduce overgrazing pressure to efficiently protect grasslands, then try to find an alternative way to maintain or increase the income of the local people [16]. Previous ecological projects on grassland management have tended to consider artifically increasing primary production, *e.g.* promoting the growth of grasses and forbs in the ecosystem. Alternatively, however, our approach considers partially replacing the major consumers of grassland ecosystems, including cattle or sheep, with less destructive animals, such as chickens [17].

Although there has been substantial government-allocated funding for the restoration of degraded grassland to projects including tree planting, fencing grassland, or rearing dairy milk cows, most of those efforts are short-lived and ineffective relative to the huge investments in the grassland [18], [19]. Based on our findings in a large-scale (2667 ha) and long-term (ten years, 2000–2009) experiment in the Bayinhushu village of the Hunshandake sandland in the northern grassland of China [16], we have proposed a novel alternative strategy which utilizes natural grasslands as an ideal place for chicken farming instead of the traditional model of raising cows and sheep. The experiment was designed to test whether chicken farming in grassland can mitagate degradation and yield more profit than traditional sheep raising. It illustrates the feasibility and advantages of chicken farming in grasslands, offering a new perspective for maintaining future grassland sustainability. Three questions are addressed: (1) Does chicken farming affect the primary production of grassland compared with unstocked areas? (2) Does chicken farming have less effect on soil water content and bulk density than traditional sheep grazing? (3) Does chicken farming increase the income of local herdsmen compared with traditional sheep grazing?

## Materials and Methods

### Study sites

The research was conducted in the Bayinhushu village of the Saiyinhuduga Sumu (Town), Zhenglan Banner (County), Inner Mongolia of China (lat 42°53.5′–42°57′N, long 116°01′ to 116°08′E, al 1150 m). The climate type varies from temperate arid to semiarid. The mean annual temperature is 1.7°C, ranging from 16.6°C in July to −24.1°C in January. The annual precipitation is about 350 mm, with an uneven summer-biased distribution over the year, whereas the potential annual evapo-transpiration is 2700 mm in the study area. Bayinhushu village is located in the Hunshandake Sandland, one of the four largest sand land areas of China. The soils are calcareous brown soils in the lowlands, with sandy soils being found in the habitats of fixed dunes, semi-shifting dunes, and shifting dunes [20]. Fixed sand dunes are dominated by *Ulmus pumila*, *Artemisia ordosica*, *Stipa glareosa*, and *Poa annua*. In semi-shifting sand dunes, *Artemisia frigida*, *Polygonum divaricatum*, and *Agropyron desertorum* are the common species. *Agriophyllum squarrosum* occurs only on shifting sand dunes.

### Experimental design

The study was conducted to test whether chicken farming can protect grassland from degradation while providing more profit than traditional sheep raising. Twelve plots (10 m×10 m) were fenced for two treatments (chicken farming and sheep raising) and the control. The control plots were free from any animals and the grasses were left to grow naturally (CK). The first treatment (T1) was designed to feed each chicken with 50 g corn per day; the second treatment (T2) was set to feed each chicken with 50 g corn and all insects caught nearby with a 314 nm UV-light lamp. The surplus corn was collected and weighed to calculate the actual amount taken by the chickens on each day. Four replicates of each treatment were randomly assigned. There were five 40-days-old male chickens in each plot. Each chicken was weighed at the beginning of experiment, every ten days during the experiment, and after 120 days.

The baby chickens were bought from the agriculture area of Shandong Province and transported to the experimental site via a special purpose vehicle on 30 April within two days after hatching in cages (60 cm×45 cm×18 cm) with holes drilled for air. The breed, “Laiwuhei chicken” is widely raised in northern villages of China. We fed them in fostering rooms where the room temperature was controlled by a circular heater. The room temperature was decreased as the chickens grew. The room temperature was set about 27–28°C for the first week, 25–26°C for the following 2–3 weeks, 22–24°C for another 4–5 weeks and 20°C for last 6 weeks. The light was kept on for 24 hours for the first week. From the second and third weeks, the light was on about 20 hours and off about 4 hours. For the following 4–6 weeks, the light time was on 12 hours and off for 12 hours. The humidity remained about 65% for the first week, then decreased to 60% and was kept constant from the second week until the sixth week. The density for the fostering rooms was about four chicken per m^2^, so there was enough space for the chickens to move around. After 40 days brooding period, we selected five male chickens with similar weight and put them in each experiment plot on 10 June.

To compare with the traditional grazing system, we set up a the third treatment (T3) of four 50 m×100 m plots to raise one sheep in each plot, based on the legally regulated numbers of sheep in one hectare. The sheep, species named “small fat-tail sheep” in Chinese, were all one-year-old males and borrowed from local herdsmen. The average weight of sheep was measured at the beginning and the end of the experiment. The control, chicken farming and sheep farming plots followed a completely randomized design.

### Plant community investigation

Plant community investigations were conducted in 1 m^2^ quadrats within each plot. Five quadrats were randomly set in each plot before the grazing treatment. Each quadrat was divided into a grid of 10 cm×10 cm cells in which the individual plants of each species were identified. Projected area on the ground of plants and the height of individual plants were recorded early in each month from June to September. Important quantitative analyses such as relative density, relative dominance, and relative abundance of species were determined according to Curtis [21]. The Importance Value Index (IVI) for species was determined as the sum of relative frequency, relative dominance and relative density. The values for species belonging to one family were summed as the importance value index of that family.

Relative density indicates the number of a species in relation to the total number of individuals of all the species, expressed as a percentage. Relative frequency is the degree of dispersion of individual species in an area in relation to the number of all the species occurred. It is expressed as the number of occurrences of the species divided by the number of occurrences for all the species. Relative dominance is determined by the value of the basal cover, which is the coverage value of a species with respect to the sum of coverage of the rest of the species in the area.

### Aboveground and root biomass measurement

After investigation of the plant community composition in September, plant aboveground and root biomass were determined. All plants were harvested and aboveground biomass was weighed fresh, and a subsample dried at 65°C for the determination of dry weight. After the harvest, three soil cores of 8 cm diameter were taken randomly in each quadrat to a depth of 50 cm and divided into four soil layers (0–10 cm, 10–20 cm, 20–30 cm and 30–50 cm). Samples were kept frozen in plastic bags before being washed. The samples were first soaked overnight (16 h) in a sodium hexametaphosphate solution (100 g L^−1^). Thereafter, root separation was performed using a hydropneumatic elutriation system [22] on a 760 µm sieve, as recommended by Boehm [23]. The roots collected on this sieve were then transferred onto a 410 µm sieve and thoroughly washed again with water to remove fine mineral particles. After this second washing, the remaining sand particles and organic debris were separated from roots by flotation. Any light organic debris mixed with roots was isolated from the roots by hand. No attempt was made to separate live and dead roots. The root samples were dried at 55°C to constant weight. The root dry weight was determined. The root: shoot ratio (R: S) was calculated by dividing the root biomass in the 0–50 cm layer by the aboveground biomass.

### Soil water content and bulk density

After the plant harvest, four soil cores of 5 cm diameter were taken randomly in each quadrat to a depth of 20 cm and divided into two soil layers (0–10 cm, 10–20 cm). The samples were put into aluminum weighing tins and brought into the laboratory. Soil water content (SWC) was determined using the gravimetric method and expressed as the mass ratio of water to dry weight, determined after oven drying at 105°C to constant weight. Three soil bulk density samples were taken in each quadrat to a depth of 20 cm and divided into two soil layers (0–10 cm, 10–20 cm). Bulk density was measured by weight of the soil per unit volume (g ml^−1^), after oven drying at 110°C.

### Assessment of chicken mass production and economic efficiency

The chickens were weighed every ten days after the experiment commenced. Relative growth rates were calculated as the gross mass increase per gram forage per day, with units of g g^−1^ d^−1^. The amount of feed was recorded for each treatment, and the feed conversion rate was the mass of food consumed relative to the body mass gain over 10 days [24]. Meat content for chickens were calculated as 70% of the gross weight, with the equivalent for sheep calculated as 60% of the gross weight. Finally, the monetary inputs for chickens and outputs for chicken and sheep grazing involved in each treatment were separately calculated to estimate the economic efficiency, which was compared with the control. The data of chicken mass from an agricultural area was provided by Wuyun [25], who followed a comparable method to that used in the grassland. This study was carried out in strict accordance with recommendations in the Guide for the Care and Use of Laboratory Animals of the Chinese Academy of Sciences. The protocol of feed animals was approved by the Chinese Academy of Sciences (Permit Number: KZCX2-YW-Q1-13). There was no surgery performed on any animals during the experiment. Following the experiment, we sold the chickens to a local business man who collected and slaughtered the chickens; and the sheep were returned to the herdsman.

### Statistical analysis

Analyses of variance were performed with SPSS version 16.0. Data for the importance value index, relative growth rate, and feed conversion rate were transformed by taking their natural logarithms to stabilize heterogeneous variances for statistical analysis. However, mean values quoted in the text have been back-transformed to the original scale. Differences in the aboveground and belowground plant biomass in grassland under chicken farming and traditional sheep grazing were tested with one-way analysis of variance (Duncan test) at p<0.05.

## Results

### The importance value index of plant communities

**Figure 1:**
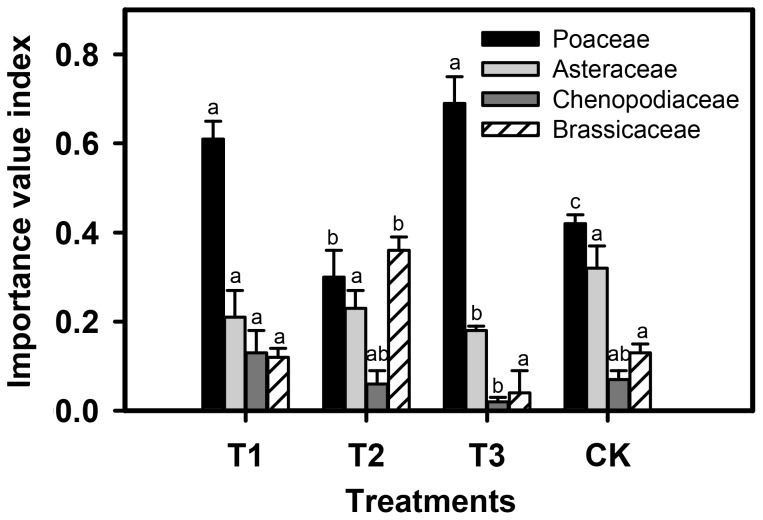
Effects of grazing treatment on the importance value index of species belonging to four plant families: Asteraceae, Chenopodiaceae, Poaceae and Brassicaceae. Values are mean ± SE (n = 4). Columns with different letters indicate significant differences at p<0.05.

The grazing treatment did not affect the number of species in plant communities, however, it had a statistically significant impact on the importance value index (IVI) of several major families. In contrast with the control, the traditional sheep grazing significantly increased the IVI of the Poaceae family (p<0.01), but decreased the IVI of the Asteraceae (p<0.001) and the Chenopodiaceae families (p<0.05). In addition, chicken fed with corn significantly increased the IVI of Poaceae (p<0.05), whereas those fed with both insects and corn enhanced the IVI of Brassicaceae (Fig. 1), implying the selective foraging of animals, *i.e.*, chicken vs sheep, and that a protein supplement in the form of insects changed chicken foraging behaviour.

### Plant biomass and allocation

**Figure 2:**
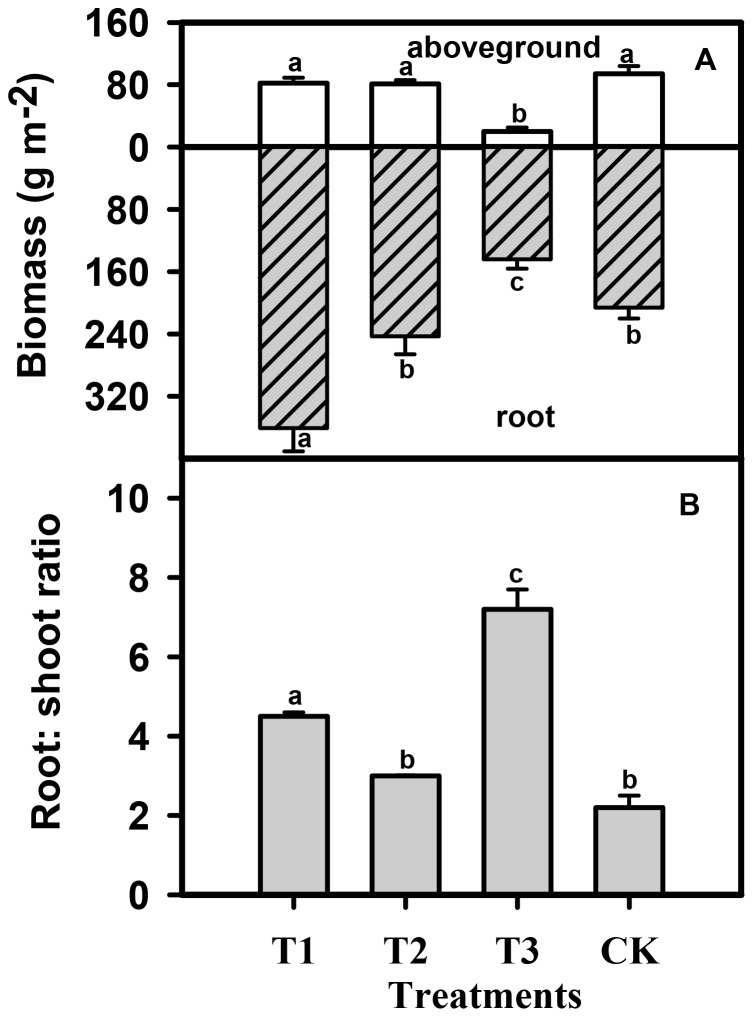
Effects of grazing treatment on aboveground and belowground plant biomass (A) and root: shoot ratio (R : S) (B). Values are mean ± SE (n = 4). Abbreviations T1: chicken fed with corn, T2: chicken fed with both corn and insects, T3: traditional sheep grazing, CK: the control without grazing. Columns with different letters indicate significant differences at p<0.05.

Chicken farming in grassland caused a significant increase in primary production in comparison with traditional sheep grazing (p<0.01), with the former yielding three times the aboveground, and twice the root biomass, of the latter (Fig. 2A). In comparison with the unstocked and fenced grassland, chicken farming did not significantly decrease aboveground production, but significantly enhanced the accumulation of root biomass (p<0.01), with an increase of 60% over that of the fenced control grassland. Root biomass was not significantly influenced by chickens fed with both corn and insects in comparison with the control. This result clearly implied that the chicken fed with both corn and insects had little impact on grassland productivity. The association of aboveground biomass and root biomass was analyzed to determine whether the grazing pattern impacted the partitioning of biomass. Against the control, the values of root to shoot ratio have been significantly increased, by 7.2 and 4.5 respectively in the sheep grazing and chicken farming systems (p<0.001). The chicken fed with both corn and insects, however, did not cause any shifts in the biomass partitioning pattern (Fig. 2B).

### Soil water content and bulk density

**Figure 3:**
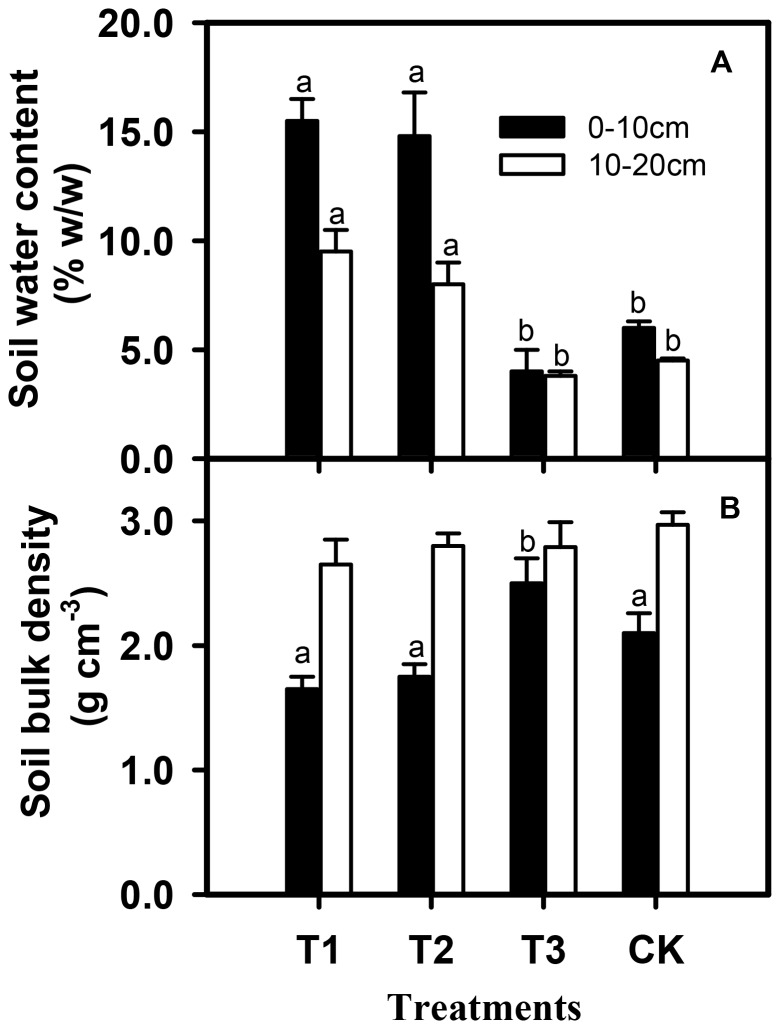
Effects of grazing treatment on soil water content (0–20 cm) (A) and soil bulk density (B). Values are mean ± SE (n = 4). Abbreviations for treatments are defined in Figure 1. Columns with different letters indicate significant differences at p<0.05.

Soil water content of the 0–10 cm layer was three times higher in grassland used for chicken farming than that used for traditional sheep grazing, and double that of the control (p<0.001) (Fig. 3A). A similar trend was noted in the 10–20 cm soil layer (Fig. 3A). Chicken farming did not affect the soil bulk density in contrast with the control. While traditional sheep grazing significantly increased the soil bulk density in the 0–10 cm soil layer by 35% of the control (p<0.05), it had no effects in 10–20 cm soil layer (Fig. 3B).

### Chicken mass production and economic efficiency

**Figure 4:**
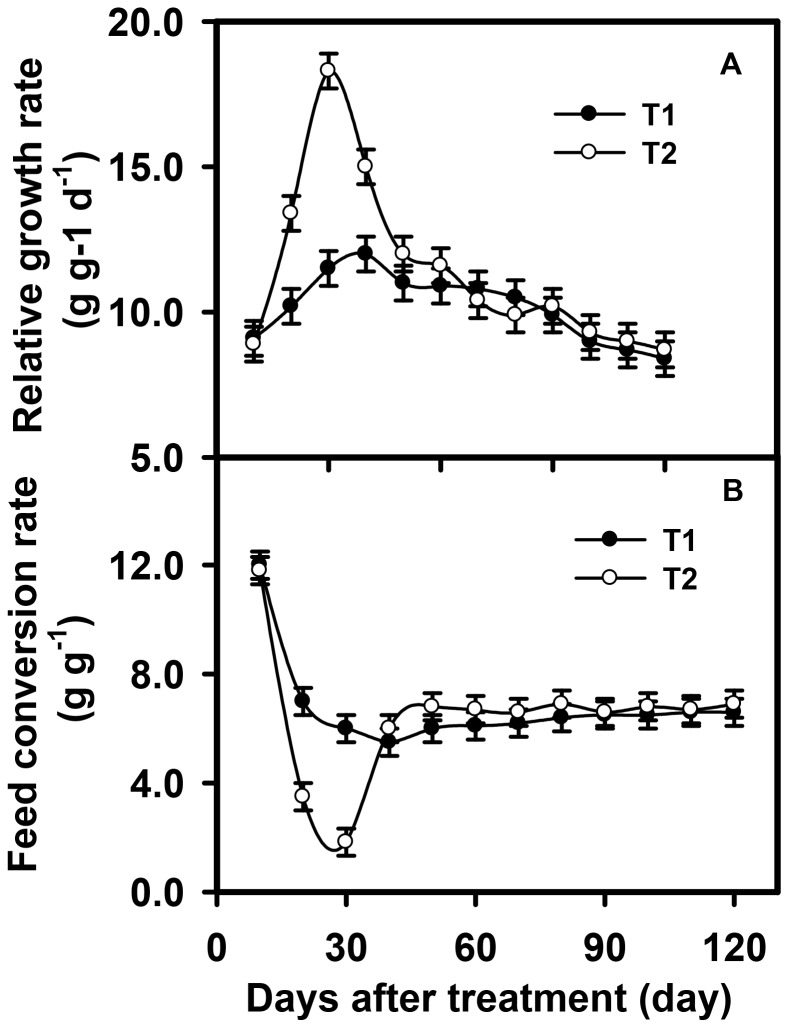
Effects of grazing model on relative growth rate (A) and feed conversion rate for chicken (B). Values are mean ± SE (n = 4). Abbreviations: T1: chicken fed with corn, T2: chicken fed with both corn and insects.

The relative growth rate of chickens did not significantly change as the experiment progressed, in spite of some fluctuations for those birds fed solely with corn (Fig. 4A). For the chickens fed with both corn and insects, the relative growth rate increased significantly from 10 to 30 days after the treatment (p<0.001) (Fig. 4A), because there were many insects available to catch. However, the amount of insects began to decrease after 40 days of the experiment, resulting in a decrease in the relative growth rate (Fig. 4A). The feed conversion ratio for the individual chickens fed with corn and insects was lower than that for birds fed solely with corn (p<0.05) (Fig. 4B).

**Figure 5:**
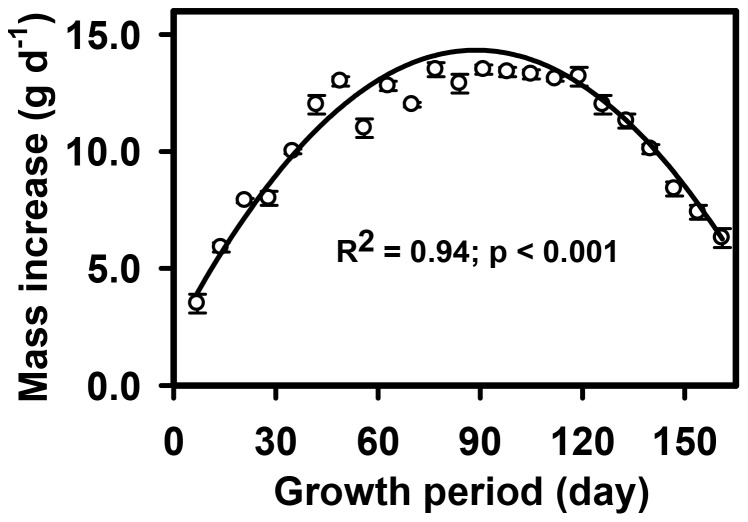
The relationship of mass increase across the whole growth period for chickens in grassland fed with corn only. The mass increase for chickens are shown as the mean ± SE for each individual (n = 4) (p<0.001; R^2^ = 0.94; y = −0.0759x^2^+1.9264x+2.1147).

**Figure 6:**
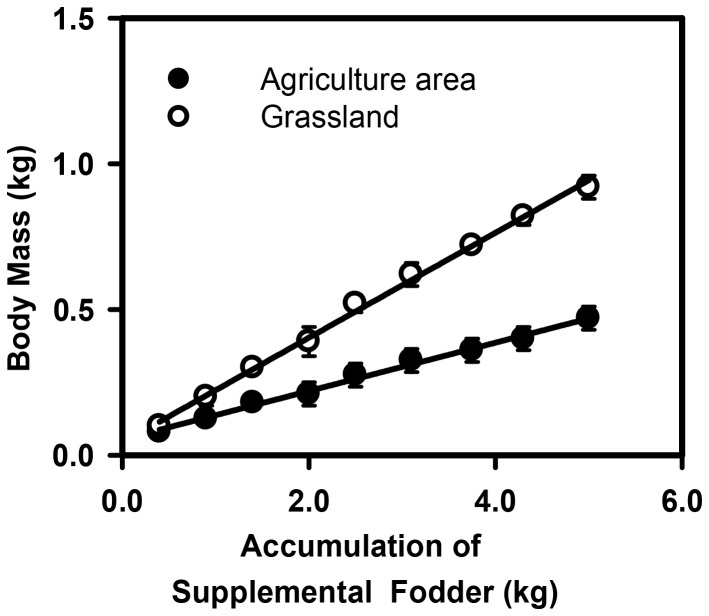
Relationship between the amount of supplemental fodder and relative weight gain for free chicken farming in a grassland ecosystem (○) and an agricultural ecosystem (•). The relationship begins at the end of the 40 day brooding stage. The average body mass of chickens after brooding is about 0.09 kg, so relative units for body mass were used. The relative weight gain for chicken was shown as the mean ± SE for each amount of supplemental fodder (n = 4) (p<0.001). The data for the agricultural ecosystem come from Wuyun.

The growth rate of the chickens fed with corn increased sharply from birth to 40 days, and showed some fluctuations from 40 to 70 days as they began to adapt to the grassland habitat (Fig. 5). From 70 days to 130 days, the growth rate tended to be stable with an average value of 11.8 g d^−1^. However, chicken growth slowed down after 140 days because of declining air temperatures. The relationship between growth rate and growth period was significant with a quadratic fit (p<0.001; R^2^ = 0.94; y = −0.0759x^2^+1.9264x+2.1147) (Fig. 5). In comparison with sheep grazing, chicken farming using supplemental corn in grassland accumulated more body mass per unit time and fodder (Fig. 6). In terms of economic efficiency, chicken farming showed the greatest economic efficiency, yielding a six-times greater return than that of traditional sheep grazing, as both chickens and hay could be sold (Table 1).

**Table 1:** Economic benefits of chicken farming, traditional sheep grazing and non-grazing per hectare of grassland.

	Parameters	Sheep grazing	Chicken farming (fed corn)	Chicken farming (fed corn+insects)	Control
Input	Corn amount (kg)	0	2615a	2418a	0
	Cost of corn (US$)	0	392.3a	362.7a	0
	Cost for immunization and hatching (US$)	0	1109.5	1109.5	0
	Cost of UV light (US$)	0	0	115.3	0
Output	Body mass increment of animals/chicken (kg)	20.8±0.9a	510.9±0.7b	520.5±0.6b	0
	Income from animals/chicken (US$)	246.1a	3024.5a	3081.4a	0
	Harvestable plant biomass (×10^3^kg)	0	0.82±0.6a	0.81±0.7a	0.94±0.4a
	Income from hay (US$)	0	59.9a	59.1a	68.6a
Net output	Income of herdsmen (US$)	246.1a	1582.6b	1644.7b	68.6c

Data for chicken farming were up-scaled to one hectare based on the data collected from our experimental 100 m^2^ plots; data for traditional sheep grazing were collected in 5000 m^2^ experimental plots. The input refers to the costs for purchasing corn, immunization for chickens and the cost of UV-lights, excluding environmental costs and labor. The outputs include the sales of hay produced from the grasses and of the animals/chicken.

Notes:The income for all products was calculated based on the local market price in 2010. The price of chicken was 5.92 US$ per kg (1.0US$ = 6.76 Chinese Yuan); the lambs were 11.83 US$ per kg; the price of hay was 0.073 US$ per kg; the price of corn was 0.15 US$ per kg. The life span of UV-light lamps was assumed to be five years and the average input was 23.6 US$ for each year. Means followed by different letters were statistically different among treatments at the p<0.05 level.

## Discussion

Chicken farming displayed considerable effects on the plant family importance in plant community by comparison with the traditional sheep grazing, indicating distinct selective foraging from different animals (birds and mammals) (Fig. 1). Nevertheless, chicken farming utilizes the grasslands only in the growing season. The precipitation is 250–370 mm in the Hunshandake sandland, with 80% of the precipitation concentrated in June and August, when there is sufficient sunlight, appropriate temperatures and humidity for grass growth. According to Glantz [26], climate can be a resource which can be exploited for society's advantage. In grassland, particularly, we tested the hypothesis that the climate is an ideal resouce suitable for healthy poultry production. The chickens were hatched during April in brooding houses, moved into the open grassland from early of June, and slaughtered in early October when the grasses have begun to senesce. Thus chicken farming in grassland avoids the traditional problems associated with larger stock animals like cattle, goats and sheep, that must use their fat as energy to maintain body temperatures during winter, resulting in low fat reserves in breeding livestock by spring. The total period of chicken farming in grassland was about four months, which greatly reduced the labor intensity expenditure of local people. Herdsmen would take a much longer time to intensively rear lambs and calves in the traditional grazing system.

The advantage of chicken farming rather than livestock in grassland has been demonstrated by the maintenance of similar aboveground plant biomass under chicken farming and the unstocked control as we expected (Fig. 2A). There are a variety of trees, shrubs, forbs and grasses in grassland, with fruits, leaves and insects forming the natural diet for free-range chickens. In order to understand how much corn feed can be saved by chicken farming in grassland, we set up an experiment in the croplands of Shandong province based on the same amount of supplemental fodder [25]. The results demonstrated that chicken weight gain with the same supplemental fodder was statistically greater in those raised in grassland than those in cropland (p<0.001)(Fig. 6). If chickens are moved from one fixed plot to another every three weeks, chicken farming will save more feed (unpublished data).

When fed with a supplemental source of protein in the form of insects, the chickens had no additional effect on the root: shoot ratio of grassland plants. This result is consistent with the hypothesis that birds cause less soil disturbance through pecking and scratching, and less compaction through trampling. Chickens cause less damage to soil by trampling than large or middle-sized mammals, which is evidenced as the increased soil bulk density of traditional sheep grazing over the control or under chicken farming (Fig. 3B). Meanwhile, supplemental feed for chickens, *e.g.* grains from agricultural areas, after turned into manure, can fertilize grassland and promote elemental cycling. Soils manured by chickens in our experiment held significantly more water than the controls or those manured by livestock, since the water-holding capacity of the sandy local soils depends critically on organic matter content. Chicken farming therefore sustains rather than degrades grassland soils. Since 2005, we have tested this proposition by experimenting with chicken farming in small grassland plots containing water sources, while leaving large degraded grasslands to be restored naturally [19]. Thus, we have simultaneously achieved full use of the natural spaces in grassland for food production, whilst reducing further degradation.

The most critical issue, however, is that most previous ecological management strategies in grassland failed to consider the economic benefits for local herdsmen. Chicken farming might be an alternative ecological restoration pathway that greatly enhances the income of local people compared with traditional grazing. Simultaneously, the naturally restored grasses can be sold as hay, thus further increasing the income of the local population. The grassland was equally divided among people based on local population and grassland productivity, making an average of 30 ha grassland for one person in the study area. Equal-sized families will therefore possess a comparable area of grassland and, generally, there are four to five people per family in local communities. In 2010, in Bayinhushu village where our experiment was conducted, for example, a five-person family raised 5000 chicken instead of sheep and cattle, and earned 4760 USD merely by selling hay. This income from hay alone was equivalent to 85% of a local family's typical annual income. Our study indicated that the economic benefit of chicken farming was about six times higher than that of grazing sheep per hectare of grassland (Table 1). Chicken farming in grassland therefore establishes the sustainability of Inner Mongolia communities from both economic and ecological points of view.

Severely degraded grassland can be revegetated via natural processes, and basic ecological functioning can be recovered within the first three years of fencing [27], significantly reducing dust storms and other hazards. Our restoration approach has been applied widely across the rest of the 10 800 km^2^ of the Hunshandake sandland in the Zhenglan county. Ecologically, a restored grassland sequesters more carbon than the degraded ecosystems that result from the traditional mode of land-use [28]. When chicken farming is integrated with this natural process of restoration, we are convinced that it represents an innovative approach to utilize grassland as a high quality organic poultry production system. China is currently raising 4.7 billion chickens, with an annual demand of 3.7 chicken per head per year [29]. However, chickens are generally raised in a crowded environment by large-scale confinement, with hormones used to promote rapid growth (45 days for the total life cycle). Feeding operations in such living conditions stress the chickens' immune systems and make them susceptible to infectious diseases. Hence, farmers have to rely on medical treatments to improve the survival rate of the birds. Unhealthy chicken, even dead ones, continue to enter the food chain due to ineffectual monitoring systems [30]. Theoretically, the omnivorous diet of chickens suits them well to farming in grassland systems because there are various natural foods available, *i.e.* insects, grasses, leaves, fruits and seeds.

**Figure 7:**
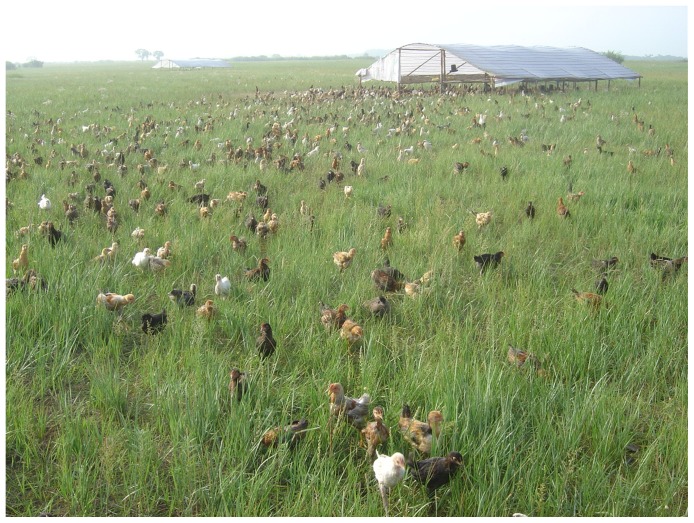
Free-range chickens reared in grassland are attractive to urban consumers as an organic food.

Grassland provides ample space for animals to range freely. However, in last decades, animal and human populations have increased sharply. The primary production in Inner Mongolian grassland is about 2.0 Mg ha^−1^ in fenced areas which are protected from animals [31]. In Bayinhushu village, there is an average 30 ha grassland per person. The current income from traditional husbandry is low, standing at only 20–50 US$ ha yr^−1^. Therefore, based on our experimental data, we propose raising chicken in relatively small areas of land (10% of the total) with a water source, while leaving other large degraded land areas (90%) to be fenced and left for natural restoration. Since soil seed banks in these grasslands are sufficiently large to sustain revegetation [27], no more human efforts are required. By adopting this strategy, the income for local herdsmen actually increased almost double. Chicken allowed to roam freely in family farms can eat natural foods such as insects, fresh green foliage and seeds, with important animal welfare benefits; they enjoy a stress-free life, breathe fresh air, and drink clean water (Fig. 7). More importantly, moving chicken to a new area can minimize contact with wild animals or birds, reducing the risk of infectious disease transmission. Chicken litter benefits plant production and soil quality of the grassland ecosystem, causing no adverse side effects on its structure and function [32].

Inevitably, chicken farming even in a small grassland will still stimulate debates about the impacts to traditionally nomadic culture, and the changes of plant community composition due to selective feeding by chickens. Thus our approach needs further investigation to avoid any shortcomings from the changes to land use. The key idea of this alternative production approach is to limit the number of medium and large livestock, by partly raising poultry, rather than prohibiting livestock grazing altogether. This novel strategy develops a new income generation stream for local herdsmen whilst simultaneously protecting the grassland ecosystem.
